# Proteomic insights into synaptic signaling in the brain: the past, present and future

**DOI:** 10.1186/s13041-021-00750-5

**Published:** 2021-02-17

**Authors:** Yalan Xu, Xiuyue Song, Dong Wang, Yin Wang, Peifeng Li, Jing Li

**Affiliations:** Institute for Translational Medicine, The Affiliated Hospital of Qingdao University, Medical College, Qingdao University, Qingdao, 266021 China

**Keywords:** Chemical synapse, Neuroproteomics, Postsynaptic density (PSD), Protein–protein interaction (PPI), Post-translational modification (PTM), Brain disorders

## Abstract

Chemical synapses in the brain connect neurons to form neural circuits, providing the structural and functional bases for neural communication. Disrupted synaptic signaling is closely related to a variety of neurological and psychiatric disorders. In the past two decades, proteomics has blossomed as a versatile tool in biological and biomedical research, rendering a wealth of information toward decoding the molecular machinery of life. There is enormous interest in employing proteomic approaches for the study of synapses, and substantial progress has been made. Here, we review the findings of proteomic studies of chemical synapses in the brain, with special attention paid to the key players in synaptic signaling, i.e., the synaptic protein complexes and their post-translational modifications. Looking toward the future, we discuss the technological advances in proteomics such as data-independent acquisition mass spectrometry (DIA-MS), cross-linking in combination with mass spectrometry (CXMS), and proximity proteomics, along with their potential to untangle the mystery of how the brain functions at the molecular level. Last but not least, we introduce the newly developed synaptomic methods. These methods and their successful applications marked the beginnings of the synaptomics era.

## Introduction

In the nervous system, the term synapse refers to the junction between two neurons or between a neuron and its target effector cells. Chemical synapses, comprising a presynaptic terminal, a postsynaptic terminal, and the synaptic cleft between them, are specialized structures for fast and precise unidirectional signal transduction and are classified primarily according to the type of transmitters involved [1]. As the minimal computational units in the central neural system, synapses interconnect billions of neurons into neural circuits, playing a crucial role in neuronal communication and functioning of the brain. Growing evidence has implicated synaptic abnormalities in various brain disorders, including psychiatric, neurodevelopmental, and neurodegenerative disorders [2, 3].

Proteomics is the characterization of all of the proteins in a biological system, such as an organelle, a cell, a tissue, or even an organism. Classified by research perspectives, there are expression proteomics, structural proteomics, and functional proteomics. Gel-based proteomics studies have been flourishing for a while [4, 5], and beginning from this century, liquid chromatography–mass spectrometry (LC–MS)-based shotgun proteomics studies emerged, with advantages such as robustness, high throughput, low labor requirement, and compatibilities with hydrophobic proteins and labeling strategies [6]. After two decades of rapid development, tens of thousands of proteins can now be studied in a high-throughput manner [7, 8]. Proteomics has already become an indispensable research tool for protein expression profiling, post-translational modifications (PTMs) detection, protein–protein interactions (PPIs) exploration, and biomarker discovery and validation, providing multidimensional insights into dynamic physiological and pathological processes in cells [9].

Proteomics met neuroscience in 1999, when a rat brain protein database containing 210 different proteins was constructed by combining two-dimensional electrophoresis (2-DE) and matrix-assisted laser desorption ionization mass spectrometry (MALDI-MS) [10]. The concept “neuroproteomics” was proposed in 2009, with a high expectation that proteomics could explain the complexity of the brain at the molecular level, from aspects including protein expression, function, bioinformatics and clinical application [11]. Over the past 20 years, proteomic technologies have increasingly been applied in neuroscience, and several excellent reviews have covered the achievements and challenges in this area [11–16].

Here, we focus on the proteomic studies of chemical synapses for the important role they played in brain development and functioning. We scrutinize the synaptic proteome in a functional context, look into the molecular mechanisms underlying brain disorders, and discuss the current opportunities, challenges, and trends in neuroproteomics. Proteomics approaches are widely used in the search for biomarkers of neurological and psychiatric disorders; however, these studies are beyond the scope of this review, and interested readers are referred to a recent review for an overview of this research field [17].

## Mapping the synaptic proteome

Despite being small in size, the synapse is surprisingly complex [18, 19]. It contains thousands of various proteins, whose spatiotemporal expression and dynamics remain a daunting challenge for researchers. In this section, we review the discoveries made over the past 20 years regarding the synaptic proteome, with discussions about sample preparation and the molecular components of the different subsynaptic structures.

### Sample preparation

**Fig. 1:**
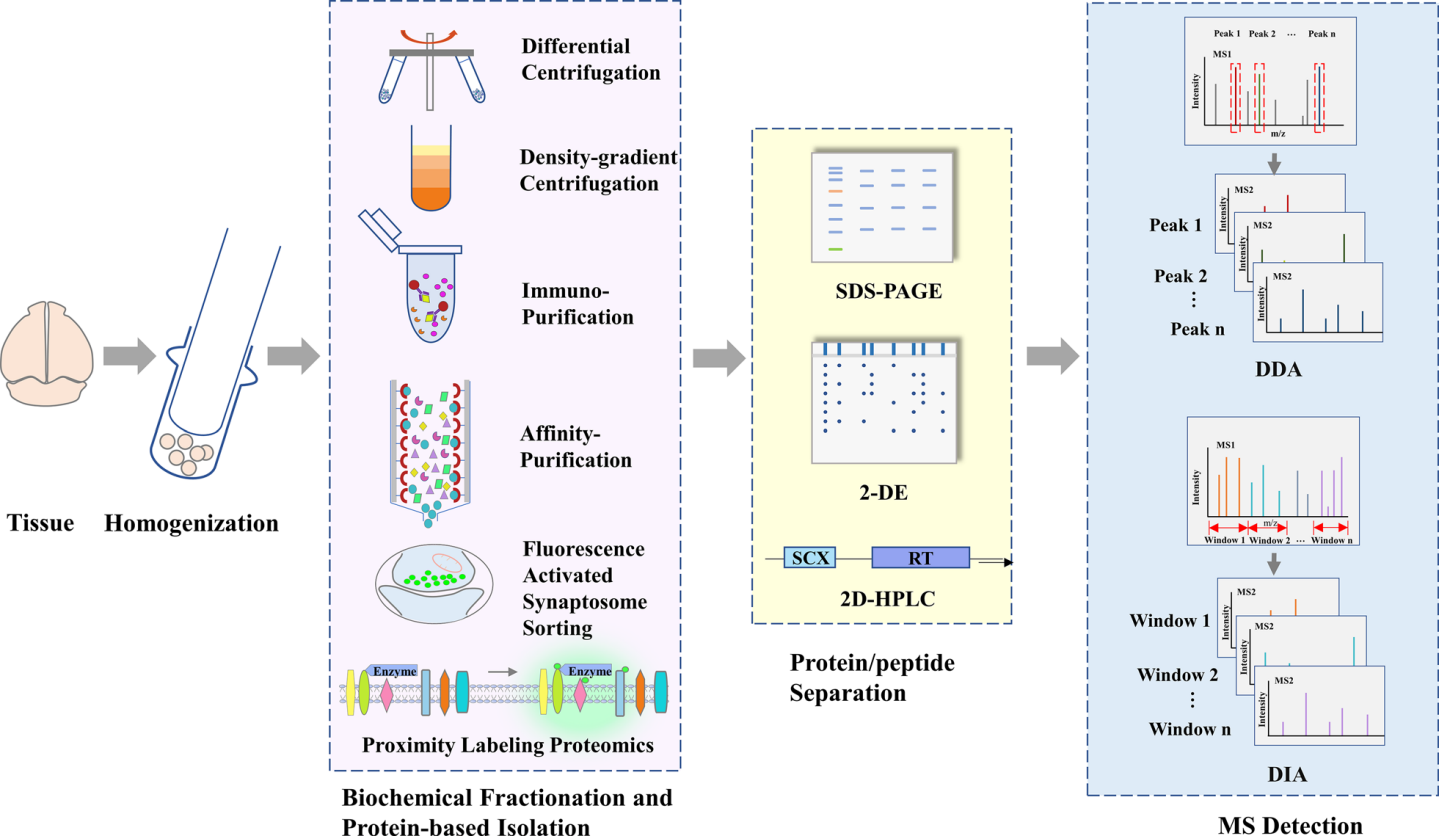
Major steps and methods in proteomic analysis of chemical synapses

A typical proteomic experiment contains major steps of sample homogenization, protein extraction, protein/peptide separation, and MS detection. In a synaptic proteomic analysis, usually the first step is to prepare synaptic samples from brain tissues or cultured cells. After being extracted from the samples, synaptic proteins can be separated by sodium dodecyl sulfate-polyacrylamide gel electrophoresis (SDS-PAGE) or 2-DE, and the protein-containing gels are cut into pieces and enzymatically digested; the other path is to digest the collected proteins first, then use chromatographic techniques, usually the combination of strong cation-exchange (SCX) chromatography and capillary reversed-phase high-performance liquid chromatography (RP-HPLC), for peptide fractionation. Usually, the last step is mass spectrometry (MS) detection: properly separated peptides were sent to MS for identification and/or quantification. Data-dependent acquisition (DDA) and data-independent acquisition (DIA) are two modes used for data collection in MS-based proteomic analysis, which we’ll discuss later. Figure 1 illustrated the major steps and methods commonly used in proteomic analysis of synapses.

The characterization of subcellular compartments relies primarily on the capability of subcellular fractionation techniques to reduce sample complexity and detect low-abundance proteins [20, 21]. One of two approaches is typically used to prepare synaptic samples: the biochemical fractionation approach utilizes differential and density-gradient centrifugation methods to isolate synaptic vesicles or artificial vesicles derived from synapses based on their unique density, while the protein-based approaches, including immunoisolation [22–25], affinity purification [26], fluorescence-activated synaptosome sorting [27] and proximity labeling methods [28, 29], isolate synaptic protein complexes or assemblies associated with the targeted proteins (Fig. 1). Both approaches are widely used and have their advantages. Generally speaking, biochemical fractionation enriches synapse-related vesicles with similar biophysical and biochemical properties, while the protein-based approaches have higher selectivity and can discriminate subtypes of synaptic vesicles [27] or synaptic assemblies derived from different types of synapses [22, 23].

### Synaptosome

The term synaptosome, created in 1964, refers to the membrane-bound sac containing vesicles that separate from synapses when brain tissue is homogenized under certain conditions [30]. As the synaptosome preserves all the main structural features of the nerve terminals, is relatively homogeneous, and has physical properties resembling those of other subcellular organelles, it has become an important window to examine the molecular machinery of the synapses. In 2003, a study combined MS with several separation techniques, including SDS-PAGE, SCX chromatography, and capillary RP-HPLC, to study the synaptic plasma membrane proteins. The authors identified several relevant synaptic proteins, including various transporters, receptors, ion channels, and enzymes, showing the effectiveness of this multidimensional separation method in profiling the proteome of membrane-associated organelles [31]. The scope of synaptosome proteomics has since expanded from comparing protein components of different synaptic vesicle pools [25] and screening synaptosome subtypes [27] to identifying long-lived synaptic proteins [32] and age- and brain region-specific changes in synaptic protein expression in animal disease models [20], which have greatly enriched our understanding of the molecular basis of synaptic signaling.

### Presynaptic components

The presynaptic terminal is the distal termination of an axon where neurotransmitters are released. The function of the presynaptic terminal is precisely regulated and is associated with brain function and diseases such as Alzheimer’s and Parkinson’s [33–35]. Integrated proteomics and systems biology approaches have been applied to obtain the molecular map of this neuronal sub-compartment, resulting in a core list of 117 proteins and 92 predicted presynaptic proteins [35]. The presynaptic active zone (PAZ) is a specialized region of the presynaptic plasma membrane, where synaptic vesicles dock and fuse and neurotransmitters are released by exocytosis [36]. Using antibodies against synaptic vesicle protein 2, researchers were able to isolate and analyze the presynaptic compartment containing the PAZ, leading to the identification of 240 proteins, including synaptic vesicle proteins, adhesion molecules, and proteins involved in intracellular signal transduction [24].

The presynaptic proteome becomes dysregulated under certain disease conditions. For example, the expression of several presynaptic markers is reduced in the hippocampal synaptic proteome of mice with a cognitive defect, consistent with the evidence of compromised presynaptic morphology and abnormal synaptic function in this brain region [37]. Proteomics approaches have also been used to reveal the proteins in presynaptic mitochondria that are important during brain development. Specifically, two independent quantitative proteomics approaches, i.e., the sequential window acquisition of all theoretical fragment ion mass spectra (SWATH-MS) and stable isotope labeling by amino acids in cell culture (SILAC), identified 40 mitochondrial proteins that are differentially expressed in mice between postnatal day 7 and day 42, including MitoNEET (Cisd1), which was revealed as a key regulator of mitochondrial function and postnatal brain development [38].

Chemical synapses rely on synaptic vesicles for signal transmission. A review of proteomic studies of synaptic vesicles published in 2007 compared different methods for synaptic vesicle separation and characterization, and highlighted the importance of analyzing subpopulations of synaptic vesicles [39]. A quantitative comparison of glutamatergic and GABAergic synaptic vesicles revealed that only a small portion of their proteins differ from each other (50 of > 450) [23]. Consistent with this result, quantitative comparison of glutamate- and GABA-specific docking complexes revealed only a few proteins that were differentially enriched in these two types of synapses, indicating high conservation of the core molecular machinery of the presynaptic docking sites [22].

### Synaptic clefts

The synaptic cleft is the space between pre- and postsynaptic terminals, which is now considered a structurally and functionally integral compartment of the synapse. Although the inability to biochemically isolate the clefts make the characterization of this structure challenging, the emerging proximity labeling proteomics approaches bring opportunities to explore the protein composition and changes in this specialized compartment. By peroxidase-mediated biotin labeling methods, researchers revealed that numerous ion channels, G protein-coupled receptors, adhesion proteins, and transporters reside in the synaptic cleft along with novel cleft candidates, and uncovered differences between excitatory glutamatergic and inhibitory GABAergic synaptic clefts [28, 29]. These studies not only demonstrated the power of proximity labeling approaches for parsing the molecular properties of traditionally unpurifiable structures, but also enriched our understanding of synaptic signaling and inspired biological hypotheses (e.g., the potential role of Mdga2 as a specificity factor at inhibitory synapses [29]).

### On the postsynaptic side

The main function of the postsynaptic terminal is to receive signals from the presynaptic terminal and transduce them to intracellular electrical and biochemical signals, thus activating downstream cascades leading to various cellular processes.

The postsynaptic density (PSD) is a complex, dynamic specialized region of the excitatory synapse that is observed as an approximately 30 nm-thick electron-dense membrane-associated structure under an electron microscope [40]. Over the past few decades, effort has been made to obtain a comprehensive picture of this protein-dominant compartment, with more than 2100 proteins identified and quantified [41–48]. The reported PSD proteome comprises various neurotransmitter receptors, cell adhesion molecules, signal enzymes, scaffold proteins, cytoskeleton proteins, and membrane transporters. The PSD composition of excitatory synapses is highly conserved between mice and humans, but the abundances of some key proteins differ, including that of some receptors and adaptor proteins that are reported to be involved in synaptic plasticity [49].

Many of the PSD proteins have been implicated in a wide range of brain disorders [50, 51]. A study in 2011 isolated PSD from human neocortex and identified 1,461 proteins [52]. Bioinformatic analysis revealed that mutations in these proteins were related to 133 neurological and psychiatric diseases and were particularly enriched in neural phenotypes involving cognition and motor functions [52]. Moreover, 143 of 700 proteins identified from PSD fractions of anterior cingulate cortex were differentially expressed in patients with schizophrenia, implicating NMDA-interacting and endocytosis-related proteins in the pathophysiology of schizophrenia [53]. These authors also identified 288 differentially expressed proteins in bipolar disorder, highlighting the important role of synaptic function and energy pathways in psychiatric diseases [54]. The role of PSD components in neurodevelopmental and neurodegenerative disorders has also been investigated [55, 56]. Nevertheless, our current understanding of this highly complex and dynamic structure remains limited and much work is still needed to elucidate its role in various pathological processes. As shown in a meta-analysis of mental disorder-related neuroproteomic studies, identification of key neural pathophysiological pathways was hindered by the poor reproducibility among independent experiments, and there were at least two reasons for this problem: one was technical reasons common to all proteomic experiments and the other was non-technical factors that specific to brain studies, including the high level of heterogeneity in the molecular pathophysiology of mental illnesses and the high biological variability found among human samples [57]. While it’s impossible to change the intrinsic features of biological samples, there are things doable to address the challenges, e.g., to use much larger number of samples (especially when working with human samples), more replicates, highly standardized procedures and detailed reports of parameters throughout the entire experiment from sample collection to data processing and report.

### Inhibitory synapses

Normal functioning of the brain depends on the orchestration of excitatory and inhibitory synapses, and an imbalance between them is implicated in many neuropathological processes [58]. Excitatory and inhibitory postsynaptic assemblies differ in protein components, molecular organizations, and biological functions [59]. However, compared with the number of studies on excitatory synapses, there are relatively few studies on inhibitory synapses. An early study once reported that inhibitory synapses lack cell signaling proteins because they didn’t find any in their study of inhibitory synaptic complexes isolated by affinity purification [26]; however, a later study using a more sensitive proximity-labeling approach has revealed that there is a huge and elaborate protein network that reside at inhibitory PSD and mediates postsynaptic inhibition [60]. This network was composed of a wide variety of proteins including neurotransmitter receptors, adhesion molecules, scaffold proteins, transporters and cytoskeletal regulators, and shared a number of signaling proteins such as Cnksr2 and Arhgap39 with excitatory PSD, suggesting a much higher complexity of the inhibitory synapse than previously appreciated [60].

## Synaptic proteomics in a functional context

While mapping of the proteome is an important step toward a complete understanding of synapse, investigation of the organization and regulatory mechanisms of synaptic proteins in a functional context is needed to comprehend synaptic signaling. With the inherent advantages in studying PPIs and PTMs, MS-based proteomic approaches have already become an indispensable tool in parsing the protein complexes and their dynamics in synaptic signaling.

### Synaptic protein complexes

Multiprotein complexes play essential roles in the organization and functioning of the synapse. They provide unique intracellular microenvironments for proteins to interact and for reactions to occur precisely and efficiently. This is most obvious in the PSD, as it contains a collection of scaffold proteins which become the hubs of the postsynaptic signaling network [61]. Coupled with subsequent MS identification, affinity pulldown and co-immunoprecipitation (co-IP) are regularly used methods for protein complex characterization. The Grant lab was one of the pioneers in utilizing proteomic tools to study synaptic complexes. They explored the organization and functional implications of the NMDA receptor complex [62, 63] and PSD95 complex [64], and published a series of enlightening reviews in the early days of the proteomic era that predicted the wide use of proteomic approaches in neuroscience research [65–68]. Later, complexes of other key synaptic proteins were resolved one after another, including PSD95 [69, 70], the SHANKs [71], SynGAP [72], PSD-93 [73], FMRP [74–76], and metabotropic glutamate receptors [77–80]; besides, the interactions among them mediated by some specific domains also attracted much attention [81, 82]. The interactome of GTPase-activating proteins and guanine exchange factors in PSD indicated their close association with the core scaffolding machinery of glutamate receptors, reflecting their active role in regulating GTP signaling from receptors to downstream targets [83]. Another study identified the interactomes of Kalirin-7 and Trio, demonstrating the importance of the interaction between Kalirin-7 and neuroligin-1 for normal synaptic function [84]. At the same time, other groups have expanded the focus from single protein complexes to dynamic PPI networks. In one of our previous work, we conducted a large-scale postsynaptic interactome study and identified 2876 proteins across 41 in vivo interactomes in the mouse cortex, thus presenting a spatiotemporal profile of the PSD signaling network and a glimpse of the assembly process of the PSD core network during development [61].

Although synaptic proteins have been implicated in many brain disorders and injuries [2, 51], their pathophysiology remains largely unknown. In recent years, a number of genetic research and genome-wide association studies have identified groups of synaptic proteins as risk factors in psychiatric disorders such as schizophrenia and autism spectrum disorders (ASD) [85–88], suggesting the central role of synaptic dysfunction in the etiologies of these diseases. Translation of these genetic findings into a physiological understanding of the underlying molecular mechanisms is a challenge faced by researchers, and proteomics has appeared as a powerful tool to fill the gap. For example, a comparison of glutamate synapse protein interactomes from different autism mouse models revealed specific and overlapping signal networks that molecularly distinguish the disease [89]. More recently, it was reported that PSD-93 interacts with SynGAP and mediates its ubiquitination and degradation, a process that may aggravate ischemic brain injury [73].

### PTM analysis

Versatile and dynamic PTMs are key features of the synaptic proteome, and accumulating evidence have indicated that the dysregulation of PTM homeostasis is associated with several pathological processes in the central nervous system, including neurodegenerative and neurodevelopmental disorders [90, 91]. MS-based proteomics is currently the most widely used approach in PTM analysis, shedding light on the functioning of the synaptic signaling machinery [92].

Phosphorylation endows the synaptic signaling network with high information-processing capacity and functional diversity, which are closely related to synaptic functions, including synaptic plasticity [93, 94]. It is also the most widely studied PTM, with mature phosphoproteomic procedures established over the years [95, 96]. Since the first mouse synaptic phosphoproteome map drawn in 2005 [97], much work, including ours, has been done to dissect the phosphorylation sites and changes under different physiological conditions, rendering valuable clues toward the mechanisms underlying synaptic functions [93, 98–102]. One of our previous work compared the phosphorylation status of the PSD proteins from mouse hippocampus before and after the induction of long-term potentiation, and found that the phosphoproteins regulated by long-term potentiation represented “PSD risk factors” for a number of psychiatric disorders [102]. Another study focusing on the presynaptic side used KCl to stimulate cultured hippocampal neurons and identified six temporal patterns of coregulated phosphoproteins, in which PAZ scaffold proteins showed a high level of activity-dependent phosphorylation-based regulation [98]. Phosphoproteomic approaches have been used to investigate the synaptic changes related to sleep by three groups independently, with interesting discoveries showing widespread alterations in the phosphorylation status of synaptic proteins during sleep and the profound molecular disturbances caused by sleep deprivation [99–101].

The phosphorylation status of single synaptic proteins and their subsequent functional influences have also been under investigation. The synaptic protein CASK, a member of the membrane-associated guanylate kinase family, can be phosphorylated by the kinase-like protein CDKL5 to promote synaptogenesis [103]. A study of ubiquitin protein ligase UBE3A found that protein kinase A phosphorylates its T485 residue to regulate the development of dendritic spines and forestall the development of ASD [104]. The protein TAOK2, whose genetic locus was associated with ASD and schizophrenia in a copy number variation study [105], mediates PSD-95 stability and dendritic spine maturation through phosphorylation of Septin7 [106].

How the phosphoproteome is regulated with efficiency and precision is a fascinating question. A recent review summarized the phosphoproteomic studies of the PSD over the years, with a focus on the components of the phosphorylation regulatory machinery, i.e., the kinases, phosphatases, and several protein domain modules, and described the general rules of the phosphoproteome-associated PSD signaling organization [107].

Besides phosphorylation, other forms of PTMs in synapses have started to receive attention in recent years. A research published in 2019 reported that DIP2A, encoded by a gene associated with susceptibility for ASD, affects synaptic transmission and morphology by regulating the acetylation of cortactin [108]. Ubiquitination plays multifaceted roles in a number of neuronal processes by controlling the quality and abundance of various proteins and has been implicated in neurodevelopmental disorders such as ASD and Angelman syndrome [109, 110]. A quantitative proteomic study showed that the ubiquitin protein ligase UBE3A mediates the ubiquitination and degradation of phosphotyrosyl phosphatase activator and affects spine morphology through a signaling pathway involving protein phosphatase 2 [111]. Table 1 summarizes proteomic studies of some key PSD proteins, providing classified information of their subcellular locations, associated diseases, molecular functions, PPI and PTM studies, and related references.

**Table 1:** Proteomic studies of important synaptic proteins

Gene name	Associated disease^a^	Molecular function	Subsynaptic location	PPI studies	PTM studies	Brain region-specific studies^b^
GRIA1	Impaired intellectual development	AMPA glutamate receptor	Postsynaptic	[43, 112–120]	Phosphorylation [121, 122] Nitrosylation [123]	1–7 [124] 2–4 [125]
GRIA2	Neurodevelopmental disorder					
GRIA3	Intellectual developmental disorder					
GRIA4	Neurodevelopmental disorders					
GRIN1	Neurodevelopmental disorder	NMDA glutamate receptor	Postsynaptic	[63, 126–134]	Phosphorylation [93, 127, 135, 136] Ubiquitination [133]	1–4, 6, 8, 9, 10 [130]
GRIN2A	Epilepsy					
GRIN2B	Mental retardation; Epileptic encephalopathy					
GRIN2C	N/A					
GRIN2D	Epileptic encephalopathy					
GRM1	Spinocerebellar ataxia	G-protein coupled receptor for glutamate	Postsynaptic	[79, 80, 137–143]	Phosphorylation [144]	2, 3 [77]
GRM3	N/A					
GRM4	N/A					
GRM5	N/A					
GRM6	Congenital stationary night blindness					
GRM7	Neurodevelopmental disorder					
DLG1	N/A	Scaffold protein	Postsynaptic	[64, 69, 70, 145–147]	N/A	N/A
DLG2	N/A					
DLG3	Mental retardation					
DLG4	Intellectual developmental disorder					
CAMK2A	Mental retardation	Calcium/calmodulin-dependent protein kinase	Postsynaptic	[148, 149]	Phosphorylation [149–154] Carbonylation [155]	N/A
CAMK2B	Mental retardation					
CAMK2D	N/A					
CAMK2G	Intellectual developmental disorder; early infantile epileptic encephalopathy					
SHANK1	N/A	Scaffold protein	Postsynaptic	[71, 156, 157]	Phosphorylation [158]	3, 5 [159, 160] 2, 3, 5 [161]
SHANK2	Autism					
SHANK3	Schizophrenia; Phelan-McDermid syndrome					
SYNGAP1	Mental retardation	RAS GTPase activating protein	Postsynaptic	[83, 162]	Phosphorylation [72, 163, 164] Ubiquitination [73]	2 [165]
HOMER1	N/A	Adaptor proteins	Postsynaptic	[101, 166]	N/A	N/A
HOMER2	Deafness					
HOMER3	N/A					
NLGN1	Autism	Adhesion proteins	Postsynaptic	[167, 168]	N/A	N/A
NLGN2	N/A					
NLGN3	Autism					
NLGN4	Autism; Mental retardation					
NRXN1	Pitt-Hopkins-like syndrome 2; schizophrenia	Adhesion proteins	Presynaptic	[169, 170]	N/A	N/A
NRXN2	N/A					
NRXN3	N/A					

^a^Data were collected from the Online Mendelian Inheritance in Man database (https://www.omim.org/)

^b^Brain regions represented by numbers (1) Olfactory bulb; (2) Cortex; (3) Hippocampus; (4) Cerebellum; (5) Striatum; (6) Thalamus; (7) Brain stem; (8) Putamen; (9). Colliculus; (10) Hindbrain

## Trends in neuroproteomics

**Fig. 2:**
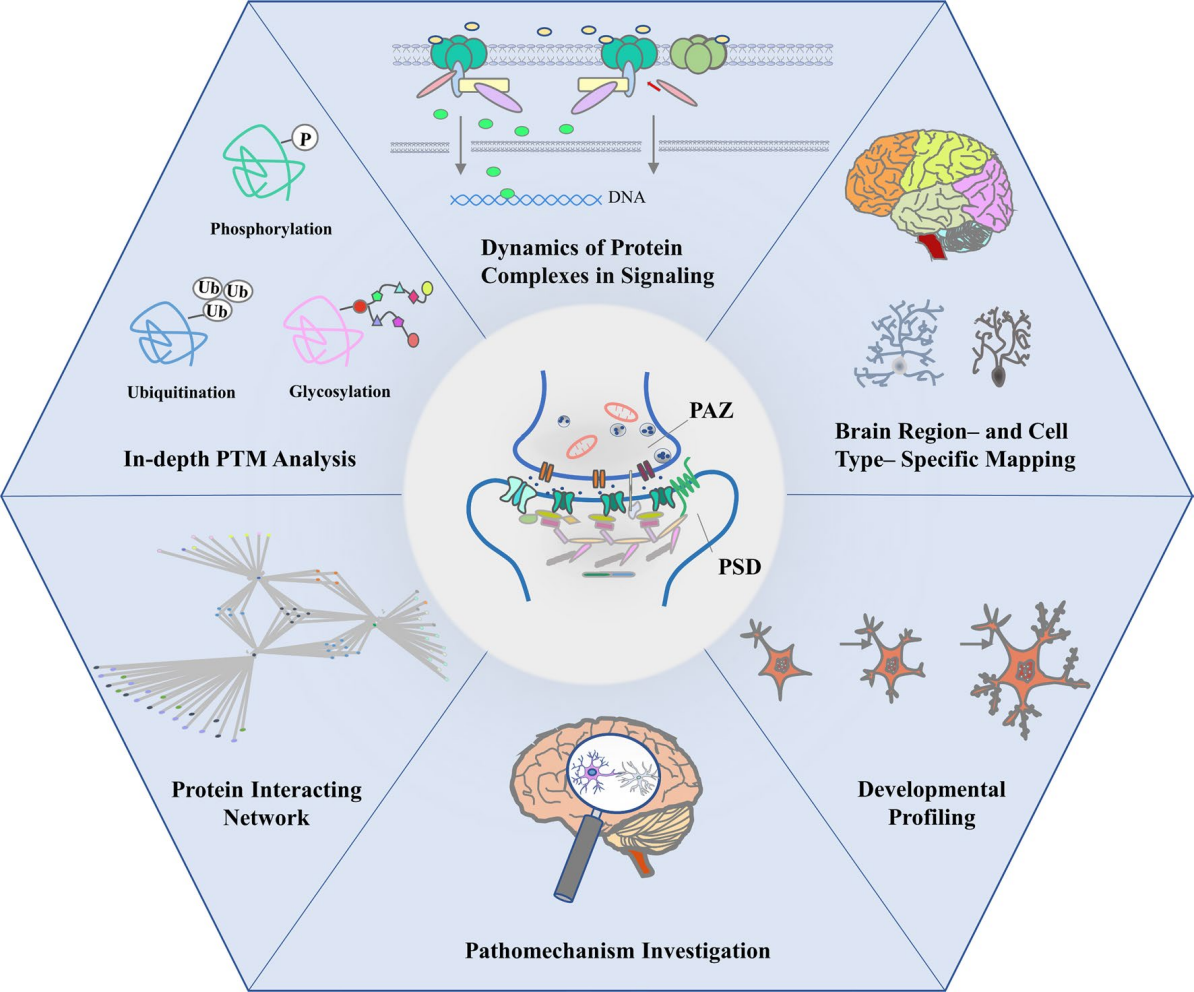
Trends in neuroproteomics

Advances in proteomic technologies have created more opportunities for deeper exploration of the brain. With the iterative upgrade of mass spectrometers and the development of novel experimental methods, including DIA, chemical cross-linking in combination with mass spectrometry (CXMS) and proximity proteomics, it will be possible to achieve in-depth analysis of protein PTMs and signaling network dynamics. At the same time, to answer the fascinating biological questions, such as the spatiotemporal landscapes of synaptic proteome and the molecular bases of various brain diseases, the great potential of proteomic tools needs to be further explored (Fig. 2). It is the interplay of advancing proteomic technologies and the emerging scientific questions that form the current trends and driving forces of neuroproteomics, leading to profound insights into neurobiology.

### Advances in proteomic technologies

#### DIA and in-depth PTM analysis

Although the importance of PTMs in cell signaling has long been recognized, our understanding of most PTMs is still in its infancy. PTMs typically feature low abundance, multiple locations, and dynamic changes, which make in-depth analyses extremely difficult. The concept of DIA was proposed in 2004 as a new MS method, in which the spectrum data were acquired based on the sequential isolation and fragmentation of preset precursor windows until a desired mass range was covered, independent of the m/z value or signal strength of the peaks [171]. In the past decade, DIA developed rapidly and has been successfully implemented in many domains of biological research [172], largely due to the development of various DIA schemes (e.g., all-ion fragmentation [173], SWATH [174, 175], multiplexed DIA [176], hyper-reaction monitoring [177], SONAR [178], and BoxCar [179]) and robust data processing tools (e.g., OpenSWATH [180], DIA-Umpire [181, 182], Spectronaut Pulsar [183], and DeepNovo-DIA [184]). Compared with the output from traditional DDA mode, DIA selects and fragments all peptides with a signal stronger than noise, rendering unbiased quantitative data with higher speed, coverage, precision, and reproducibility [185], and making large-scale in-depth PTM analyses a reality. Through targeted optimization of experimental DIA methods and algorithms, a recent study quantified more than 20,000 phosphopeptides in a 15 min liquid chromatography–MS analysis [186]. Besides phosphorylation, DIA has also been utilized to study the changes to histone PTMs induced by the lead compound acridone derivative 8a, presenting quantitative methylation and acetylation data for epigenetic analyses [187]. There is reason to believe that with the continuous improvement of experimental methods and software toolkits, sophisticated DIA workflows will greatly accelerate PTM research in the near future, becoming a powerful tool for the in-depth study of synaptic signaling mechanisms.

#### CXMS and the investigation of direct PPIs

Most proteins accomplish their tasks through interactions with other proteins. Only with a detailed understanding of a protein’s interactors can we begin to probe its function and the consequences of its mutation. Popular methods for PPI identification include the yeast two-hybrid system [188], Förster resonance energy transfer (FRET) method [189, 190], and MS-based methods such as affinity chromatography [191] and co-IP [192]. However, these methods have limitations. In the yeast two-hybrid system, the yeast cell may not provide a suitable cellular environment for in vivo PPIs that occur in mammalian cells; FRET measured by fluorescence lifetime microscopy is expensive and low throughput, which makes it most suitable for hypothesis-driven experiments; with affinity chromatography and co-IP, it is difficult to tell between direct and indirect PPIs.

CXMS uses chemical agents to link two amino acids that are within a certain spatial distance (usually 7–40 Å, depending on different cross-linkers) and can react with the cross-linker via valence bonds [193]. The cross-linked products are analyzed by MS-based proteomics to identify interactors and direct interface regions [194, 195]. The advantages of CXMS include relatively low cost, suitability for detecting transient and loose interactions, ease of excluding contaminants, and the ability to differentiate between direct and indirect interactions. The last decade has seen an increase in studies employing CXMS for protein complex analysis [196]. The first application of CXMS for the study of synaptic protein complexes was published recently, in which researchers identified nearly 12,000 unique cross-links within and between 2362 proteins [197]. Most cross-links identified here were loop links, i.e., the cross-linker reacted with two amino acids within the same protein; nevertheless, it marked the first direct PPI map of mouse brain synapses and was seen as an illuminating proof of the concept of applying CXMS in complex biological samples.

#### Proximity proteomics and the exploration of protein neighbors

Another emerging method for studying protein interactions is proximity proteomics. For this method, certain enzymes are attached to the target protein to generate small molecules, which can covalently label proteins. The short half-life of the small molecules ensures that only the close neighbors of the target protein are labeled for subsequent enrichment and analysis [198–200]. It has a larger spatial range (from ten to hundreds of nanometers) than chemical cross-linking, enabling its use for exploring weak and transient protein interactions as they occur in vivo [201, 202]. Proximity proteomics has found its arena in the study of synapses, where receptors, scaffolding proteins, kinases, cytoskeleton proteins, and various regulatory molecules are closely arranged to form a precisely controlled, highly orchestrated signaling network, and it has demonstrated its usefulness in the analysis of excitatory synaptic clefts, a subcellular compartment that cannot be isolated by traditional biochemical methods [28]. Based on the enzyme used to generate the small tags, proximity labeling methods can essentially be classified into two categories: peroxidase-based and biotin ligase-based proximity labeling [203]. A landmark study of the application of proximity proteomics in neuroscience was published in 2016, in which unique features of excitatory and inhibitory synaptic clefts was revealed by the peroxidase-based APEX proteomics [29]. In the same year, researchers from Duke University and their collaborators employed a biotin ligase-based proximity labeling approach, the proximity-dependent biotin identification (BioID) approach, to identify inhibitory postsynaptic proteins, and uncovered a functionally diverse protein assemblage that regulates postsynaptic inhibition [60]. Three years later, the same group published their study of nascent synapses by in vivo version of BioID (iBioID) and revealed a novel mechanism underlying excitatory synaptogenesis [204]. The group also shared their protocol for the iBioID proximity proteomics method [205], providing an easily adapted technique for the study of substructures that are difficult to purify. Although most proximity labeling methods are still in the early stages of application and have some limitations such as stimulations to cells or poor labeling efficiency [203], its unique advantages (e.g., not limited by the availability of antibodies, the ability to specifically label target proteins in a certain cell type when combined with conditional gene expression techniques) are increasingly being noticed by researchers. It complements classical IP methods, showing potential for a comprehensive map of synaptic PPI network.

### Appealing biological questions

#### Depicting the spatial molecular landscape of the brain

As the most complex organ in mammals, the brain is anatomically divided into different regions, each with distinct cell types and arrangements, and associated with various physiological functions and pathological processes [206, 207]. A brain region- and cell type-specific proteome map is of unique importance for understanding brain functioning and the molecular basis of related diseases. Large-scale proteome analysis of ten brain regions and cell types in mice revealed that, in line with the functional specificity, protein expression patterns showed cell type- and region-related diversity [208]. Furthermore, subregions within an area can be distinguished, such as the different protein expression profiles for CA2 and CA1 subregions within the hippocampus [209]. In 2008, a quantitative proteomic study comparing PSD preparations from the mouse cortex, midbrain, cerebellum, and hippocampus revealed that the hippocampus had the highest kinase and phosphatase contents, with relatively high overall phosphorylation levels, indicative of a large amount of synaptic signal transduction in this area [210]. Researchers have also found significant molecular heterogeneity between PSD fractions purified from rat forebrain and cerebellum [211]. A more recent study showed high regional diversity of the mouse postsynaptic proteome, with up to 74% of proteins exhibiting differential expression and a unique compositional signature in each region [212]. The functions of specific brain regions in pathological processes have also been under investigation: a team from Switzerland obtained PSD proteomes from striatal and hippocampal tissues of adult Shank3 mutant mice and revealed novel brain region-specific alterations associated with ASD risk genes [160]. Recently, the same group performed proteomic analysis of the pre- and postsynaptic compartments in different brain regions of male and female adult mice and reported new sets of the region- and sex-specific synaptic proteins [213]. Another collaborative study used Shank3B^−/−^ mutant mice to show that during the second and third postnatal weeks, the striatal synaptic proteome is extensively remodeled, implicating the abnormal maturation of striatal circuits in the behavioral deficits exhibited by these mice [214]. Regional profiling of PSD proteins has also been conducted using human neocortex samples and, by integrated analysis of genetic and proteomic data, provided relevant information to identify brain regions involved in behavioral pathology [215].

The brain comprises a complex network of various cell types and fiber bundles, and efforts have been made to reveal the protein components of specific synaptic types, such as the parallel fiber-Purkinje cell synapse [216]. Cell type-specific proteomics is an emerging frontier leading to discoveries of functional and molecular diversities, including recent analyses on brain mitochondria [217] and the psychiatric disorder-related protein DISC1 interactome [218]. Furthermore, proteins can have more than one subcellular location, executing distinct functions with different partners. Many PSD proteins form distinct multiprotein complexes inside and outside the PSD [61], indicating the need for spatial profiling on a more precise scale. The relationships and regulatory mechanisms of these complexes may be the key to understanding synapse-to-nucleus signaling.

#### Tracing the temporal changes during development

Brain development is a complex and protracted process involving the formation, strengthening, and elimination of synapses [219]. The importance of developmental analyses has long been recognized, and the first large-scale proteomic analysis of synaptic development was published in 2007, which utilized stable isotope labeling in mammals to quantitatively compare synaptic proteins in mouse cerebellum at four postnatal stages (post-natal day 1 (p1), p10, p20, and p45) of development [220]. These researchers used the same approach in a subsequent study of synaptosomal and mitochondrial fractions from three rat brain regions at four postnatal time points to identify novel regulators of neurodevelopment [221]. Other quantitative proteomic approaches, including the multiplexed iTRAQ method [222] and label-free quantitation [61], have also been utilized in the study of the synaptic proteome dynamics during rodent brain development. Findings from the spatiotemporal profiling of the PSD proteome indicated that postnatal day 7 is a key time point in synaptic development when a complete three-layer scaffold structure of PSD first forms [61]. On the timescale of lifespan, tools have been developed to identify age-dependent gene regulatory events and the results showed the postnatal developmental changes in the synaptic proteome and their relevance to the age of disease onset for specific neurological disorders, including schizophrenia [223]. More recently, the molecular and morphological features of five billion excitatory synapses were depicted across the mouse brain from birth to old age, representing the most comprehensive analysis of synapses during development and aging so far [224].

#### Unveiling the mysteries of brain disorders

Brain disorders, including developmental, psychiatric, and neurodegenerative disorders, affect individuals of different ages and represent a major challenge to human health. Proper brain function depends on orderly and controlled neuronal excitation and inhibition. As the basic connecting and computing unit in the central nervous system, synapses are crucial to all forms of brain activity and synaptic proteins had been implicated in many brain disorders [225]. Thus, the development of effective therapy for these disorders depends on an in-depth understanding of the molecular and functional organization of synapses.

Proteomics has long played an indispensable role in the discovery and verification of biomarkers for brain disorders [17], and studies are increasingly oriented towards exploring the pathological mechanisms underlying various brain disorders. In a recently published work, researchers found that the astrocytic cystine/glutamate antiporter system xc(-) regulated corticostriatal neurotransmission, and influenced social preference and repetitive behavior in mice, providing important clues toward the neural mechanisms related to ASD and obsessive–compulsive disorder [226]. Synaptic proteome abnormalities related to several psychiatric disorders have been revealed in the last decade, and a number of signaling pathways are involved [54, 160, 227, 228]. For example, GABAA receptor blockade in the hippocampus improved synaptic plasticity in an Alzheimer’s disease mouse model, leading to the identification of several proteins that contribute to learning and memory functions in this disease [229]. We have studied the spatial distribution and maturation of PSD interactomes, as well as the phosphorylation network regulated by long-term potentiation, and found that these networks converge at a number of highly connected nodes, which also represent PSD risk factors for many psychiatric disorders [61, 102]. Thus, pathological processes underlying these disorders likely involve the loss of PSD protein interactions and the subsequent dysregulation of synaptic signaling.

## Future perspectives

Recent years have seen solid progress in the research field of synapses boosted by the orchestration of genetic, genomic, and proteomic studies. In 2019, a large-scale collaborative study established the Synaptic Gene Ontology (SynGO) database, an evidence-based, expert-curated knowledge base for “discovering the synapse” [230]. It features detailed and traceable annotations of synaptic proteins, offering a universal reference and a powerful yet convenient online platform to facilitate the study of synapses.

In the latest study from one of the leading labs in the field of proteomics, researchers combined technologies such as DIA-proteomics, DIA-phosphoproteomics, proximity labeling, and cross-linking MS to reveal how a mutation in the tumor-driving gene Dyrk2 affects downstream cellular processes [231], demonstrating the great potential of multilayered proteomics for decoding the molecular machinery of life. At the same time, with continued improvements in mass spectrometers and sample processing methods, single-cell proteomics has stepped onto the stage, making more specific and accurate descriptions of molecular dynamics feasible [232]. Specific to the study of synapses, with the combination of genetic labeling and imaging methods, spatial diversity of synaptic proteins could now be seen with single-synapse resolution across all regions of the mouse brain [233, 234]. These new methods have recently been applied on a brain-wide scale to examine the protein composition of individual synapses throughout the mouse lifespan, and spatiotemporal changes of synapse composition with potential relevance to intellectual ability and behavioral disorders have been discovered [224]. These brilliant works represent the beginnings of synaptomics and will be extended with more proteins being examined in various physiological and pathological conditions, delivering new insights into our understanding of the synapses and the brain.

## Data Availability

Not applicable.

## Abbreviations

2-DE: Two-dimensional electrophoresis
ASD: Autism spectrum disorder
BioID: Biotin identification
CXMS: Chemical cross-linking in combination with mass spectrometry
co-IP: Co-immunoprecipitation
DDA: Data dependent acquisition
DIA: Data independent acquisition
FRET: Förster resonance energy transfer
LC–MS: Liquid chromatography mass spectrometry
MALDI-MS: Matrix-assisted laser desorption ionization mass spectrometry
MS: Mass spectrometry
PAZ: Presynaptic active zone
PPI: Protein–protein interaction
PSD: Postsynaptic density
PTM: Posttranslational modification
RP-HPLC: Reversed-phase high performance liquid chromatography
SCX: Strong cation-exchange
SDS-PAGE: Sodium dodecyl sulfate polyacrylamide gel electrophoresis
SILAC: Stable isotope labeling by amino acids in cell culture
SWATH-MS: Sequential window acquisition of all theoretical fragment ion mass spectra
